# Screening of the PA14NR Transposon Mutant Library Identifies Genes Involved in Resistance to Bacteriophage Infection in *Pseudomomas aeruginosa*

**DOI:** 10.3390/ijms25137009

**Published:** 2024-06-26

**Authors:** Peiying Ho, Linh Chi Dam, Wei Ren Ryanna Koh, Rui Si Nai, Qian Hui Nah, Faeqa Binte Muhammad Rajaie Fizla, Chia Ching Chan, Thet Tun Aung, Shin Giek Goh, You Fang, Zhining Lim, Ming Guang Koh, Michael Demott, Yann Felix Boucher, Benoit Malleret, Karina Yew-Hoong Gin, Peter Dedon, Wilfried Moreira

**Affiliations:** 1 Antimicrobial Resistance Interdisciplinary Research Group (AMR IRG), Singapore-MIT Alliance for Research and Technology (SMART) Centre, Singapore 117576, Singapore; peiying@smart.mit.edu (P.H.); chi.dam01@u.duke.nus.edu (L.C.D.); mdcv327@partner.nus.edu.sg (W.R.R.K.); jojo.nah@smart.mit.edu (Q.H.N.); faeqafizla@smart.mit.edu (F.B.M.R.F.); jean800624@gmail.com (C.C.C.); pcdedon@mit.edu (P.D.); 2 Signature Research Program in Cardiovascular & Metabolic Disorders, Duke-NUS Medical School, Singapore 169857, Singapore; 3 Department of Medicine, National University Hospital, National University Health System, Singapore 119074, Singapore; 4 Thrixen Pte Ltd., Singapore 048619, Singapore; 5 Immunology Translational Research Programme, Department of Microbiology and Immunology, Yong Loo Lin School of Medicine, National University of Singapore, Singapore 117597, Singapore; thet.tun.aung@seri.com.sg (T.T.A.); lzhining@nus.edu.sg (Z.L.); koh.mg@nus.edu.sg (M.G.K.); benoit_malleret@nus.edu.sg (B.M.); 6 Energy & Environmental Sustainability Solutions for Megacities (E2S2) Program, Campus for Research Excellence and Technological Enterprise (CREATE), Singapore 117576, Singapore; erigsg@nus.edu.sg (S.G.G.); ceeginyh@nus.edu.sg (K.Y.-H.G.); 7 Department of Civil & Environmental Engineering, National University of Singapore, Singapore 117576, Singapore; 8 Singapore Centre for Environmental Life Science Engineering (SCELSE), Singapore 637551, Singapore; ephyb@nus.edu.sg; 9 Department of Biological Engineering, Massachusetts Institute of Technology, Cambridge, MA 02139, USA; msdemott@mit.edu; 10 Saw Swee Hock School of Public Health, National University of Singapore, Singapore 117549, Singapore; 11 Environmental Research Institute, National University of Singapore, Singapore 117576, Singapore; 12 Life Science Institute, National University of Singapore, Singapore 117456, Singapore

**Keywords:** *Pseudomonas aeruginosa*, bacteriophage, bacteriophage resistance mechanisms, transposon mutagenesis

## Abstract

Multidrug-resistant *P. aeruginosa* infections pose a serious public health threat due to the rise in antimicrobial resistance. Phage therapy has emerged as a promising alternative. However, *P. aeruginosa* has evolved various mechanisms to thwart phage attacks, making it crucial to decipher these resistance mechanisms to develop effective therapeutic strategies. In this study, we conducted a forward-genetic screen of the *P. aeruginosa* PA14 non-redundant transposon library (PA14NR) to identify dominant-negative mutants displaying phage-resistant phenotypes. Our screening process revealed 78 mutants capable of thriving in the presence of phages, with 23 of them carrying insertions in genes associated with membrane composition. Six mutants exhibited total resistance to phage infection. Transposon insertions were found in genes known to be linked to phage-resistance such as *galU* and a glycosyl transferase gene, as well as novel genes such as *mexB*, *lasB*, and two hypothetical proteins. Functional experiments demonstrated that these genes played pivotal roles in phage adsorption and biofilm formation, indicating that altering the bacterial membrane composition commonly leads to phage resistance in *P. aeruginosa*. Importantly, these mutants displayed phenotypic trade-offs, as their resistance to phages inversely affected antibiotic resistance and hindered biofilm formation, shedding light on the complex interplay between phage susceptibility and bacterial fitness. This study highlights the potential of transposon mutant libraries and forward-genetic screens in identifying key genes involved in phage-host interactions and resistance mechanisms. These findings support the development of innovative strategies for combating antibiotic-resistant pathogens.

## 1. Introduction

*Pseudomonas aeruginosa* is one of the most problematic opportunistic pathogens and a common cause of hospital-acquired infections [1,2]. It is listed on the World Health Organization’s list of critically important antibiotic resistant pathogens, for which therapeutic options are increasingly limited. It is also a frequent cause of chronic lung infections in cystic fibrosis patients [3]. Carbapenemase-producing *P. aeruginosa* drastically limits the number of efficacious antibiotics [4]. Phage therapy (PT), that is, the use of a cocktail of natural phages targeting *P. aeruginosa*, has been considered an alternative option for multi-drug resistant cases. PT against pseudomonal infections dates back more than 50 years [5], and recent clinical successes have been reported [6,7,8,9]. However, the ability of bacteria to become resistant to phage infections combined with our limited understanding of the underlying cellular and molecular mechanisms of resistance threaten the success of phage therapy.

*P. aeruginosa* has developed multiple defense mechanisms to safeguard itself against phage attacks [7]. These mechanisms include reducing phage adsorption, inhibiting the injection of phage genetic material, and degrading the phage nucleic acids. Phage adsorption is the initial step where phages recognize and attach to the bacterial cell surface. It involves specific external features of the bacterial membrane such as the lipopolysaccharides (LPS), the flagellum, or the pili [10]. Accordingly, one of the most common resistance mechanisms *P. aeruginosa* employs against phage infection involves altering its membrane composition. In response, phages adapt by mutating their receptor-binding proteins [11]. Several reviews on phage resistance mechanisms have been published [12,13,14,15]. Nonetheless, resistance mechanisms in *P. aeruginosa* are not fully understood, and new mechanisms continue to be identified [16,17,18,19,20,21]. The comprehension of these mechanisms is important for several reasons. For one, this knowledge is critical for the design of a therapeutic cocktail of phages that would limit the emergence of resistant bacteria. Additionally, it provides insight into essential cellular mechanisms or molecules that phages require to successfully infect and replicate within their host cells [22].

Here, we use a forward-genetic approach and interrogate the PA14NR transposon library [23] for dominant-negative mutants that display phage-resistant phenotypes. We identified 78 mutants capable of growing in the presence of phages, 23 of which with insertion in genes were predicted to be involved in membrane composition. Six mutants showed total resistance to phage infection. Genetic analysis of the six mutants revealed the interruption of two hypothetical proteins, two genes previously linked to resistance (*galU* and a putative glycosyl transferase), and two novel genes required for phage infection (*mexB* and *lasB*). We used both phenotypic and genetic approaches to show that these genes are involved in biofilm formation and resistance to phage infection, predominantly through reduced phage-adsorption. We also show that the mutants exhibited phenotypic trades-off, whereby a resistance to phage reverted antibiotic resistance or impeded antibiotic-tolerant biofilm formation.

## 2. Results

### 2.1. Screening of the Complete PA14NR Library Identifies Mutants Resistant to Phage Infection

**Figure 1:**
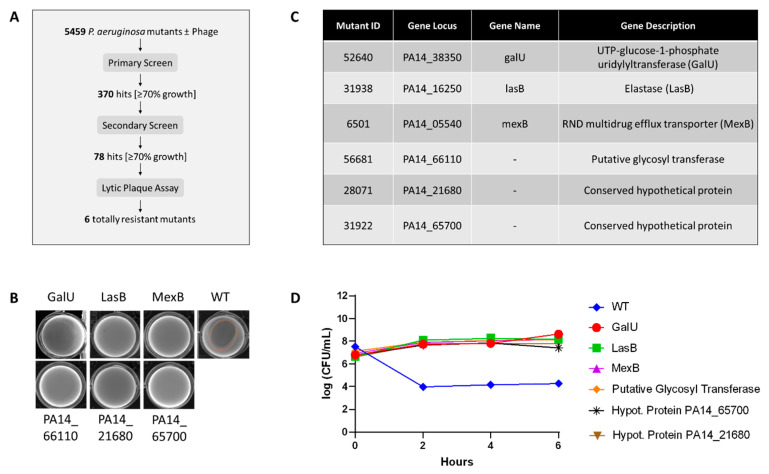
Screening of the PA14NR library identifies phage-resistant mutants. (**A**) Screening cascade of the complete PA14NR set. The library was screened in a phenotypic assay (see Section 4) where mutants were grown in the presence or absence of phages. Both screens used the same selection criteria, whereby a hit was defined as mutants with a growth value in the presence of phages superior or equal to 70% of the mutant alone. The final screen used the presence or absence of lytic plaques as a proxy for productive phage resistance. We showed that six *P. aeruginosa* mutants were totally resistant to phage infection (no plaque). (**B**) Lytic plaque assay. Phages form clear lytic plaque on upon infecting wildtype *P. aeruginosa* while no plaque is visible on any of the six mutants. (**C**) Transposon-mutant information. Mutant identification, gene locus, gene name, and gene description were obtained from the PA145NR public database and reported for each mutant. (**D**) Kill-kinetic of phages against PA14 wildtype and transposon mutants. None of the mutants showed a reduction in CFU over time as compared to wildtype. Experiments were carried out in triplicate. Representative results are shown.

To identify dominant-negative mutations that confer resistance to phage infection, we systematically screened the entire PA14NR library of 5459 transposon mutants (Figure 1A). The screening utilized a phenotypic growth assay in the presence of a single lytic phage, vB_Pae_SG_Moreira_PyoP2. This phage is a virulent tailed bacteriophage that infects P. aeruginosa, belonging to the order Caudovirales, family Myoviridae, and genus Pbunaviruses. PA14 mutants were grown either alone or in the presence of the phage. The primary screen was carried out once and yielded 370 hits. Primary hits were defined as phage-treated mutants displaying growth equal or superior to 70% of their respective untreated mutants. This threshold was selected to identify mutants whose growth ability was not hindered by the presence of phage, while also considering the growth variability under the screening conditions. Using the same criteria, the secondary screen was carried out twice and validated 77 mutants. Mutants with critical growth defects (OD_600_ < 0.05) were excluded. The drop in the number of hits between primary and secondary screening can largely be attributed to the growth variation between the primary and secondary screening, combined with the exclusion of mutants bordering the 70% cut-off. To identify mutants that do not support a productive phage infection, we carried out a lytic plaque assay on the 77 mutants. Six of them did not support plaque formation (Figure 1B and Supplementary Table S1).

The genetic background information of the all mutants was retrieved from the PA14NR database (Figure 1C). Interestingly, 23 of the 77 mutants had transposon insertions in genes involved in membrane composition or membrane components (Supplementary Table S2). Transposon insertion sites of the six mutants that did not support plaque formation revealed that mutants 31922 and 28071 carried insertions in uncharacterized conserved hypothetical proteins. Mutant 56681 carried an insertion in a putative glycosyl transferase, an enzyme involved in LPS biosynthesis. A glycosyl transferase mutant in *P. aeruginosa* strain PA1 was previously associated with defective LPS and resistance to phages [24]. The transposon insertion in mutant 6,501 was in the *mexB* gene, located in the *mexAB-OprM* operon, which encodes the RND-type multidrug efflux transporter [25]. Mex systems are typically made of three components embedded in the bacterial membrane, which actively transport molecules, including antibiotics, out of the cell [26]. MexB is an antiporter that translocates molecules from the cytoplasm to the periplasm and anchors the complex in the bacteria’s inner membrane. It is linked to OprM, an outer membrane protein that forms a surface-exposed channel through MexA, a periplasmic membrane fusion protein. OprM was previously shown to be a phage receptor and deletion of the OprM gene in *P. aeruginosa* PAO1 conferred resistance to phage while reverting antibiotic resistance [27]. Mutant 52640’s insertion was in the *galU* gene coding for the UTP-glucose-1-phosphate uridylyltransferase (GalU). This protein converts Glucose-1-phospate to UDP-D-Glucose, a predominant sugar in the outer core of LPS. A large chromosomal deletion including *galU* was previously shown to confer resistance to phage infection in the *P. aeruginosa* PA1r strain [28]. Finally, the insertion in mutant 31938 disrupted the *lasB* gene coding for the elastase LasB, a zinc metalloprotease and major virulence factor of *P. aeruginosa* [29]. We further confirmed the phage-resistance phenotype of each mutant in a time-kill experiment (Figure 1D), in which survival of PA14 wildtype and mutants in the presence of the phage was monitored over time. None of the mutants showed reduction in colony forming unit (cfu) count over time, while the phage reduced viability of the wildtype by several orders of magnitude as early as 2 h post-inoculation.

To validate that the resistance phenotype was caused by the transposon insertion in the identified genes, we cloned the wildtype sequence of each gene under the control of a strong promoter on a pBR1 plasmid backbone. Each construct was introduced in its corresponding mutant by electroporation and transformants were selected on kanamycin plates. We predicted that genetic complementation would restore sensitivity to phage killing and determined the phage susceptibility profile of the revertant by testing for lytic plaque formation. As predicted, the transformation of plasmids carrying either *galU*, *mexB*, the putative glycosyl transferase, or the hypothetical proteins into their corresponding transposon mutants restored the sensitivity to phage infection and formation of lytic plaque. However, the transformation of plasmids carrying *LasB* did not revert the resistance phenotype of the transposon mutants. (Supplementary Table S1). The results above indicate that the six mutants identified were totally resistant to infection by phage vB_Pae_SG_Moreira_PyoP2, and that in four cases it could be shown that the interrupted genes (*galU*, *mexB*, and the hypothetical proteins) mediated the resistance phenotypes. These genes have not been previously associated with resistance to phages, except for *galU* and an orthologue of the gene coding for a glycosyl transferase.

### 2.2. Phage-Resistant Mutants Exhibit Phenotypic Trades-Off

**Figure 2:**
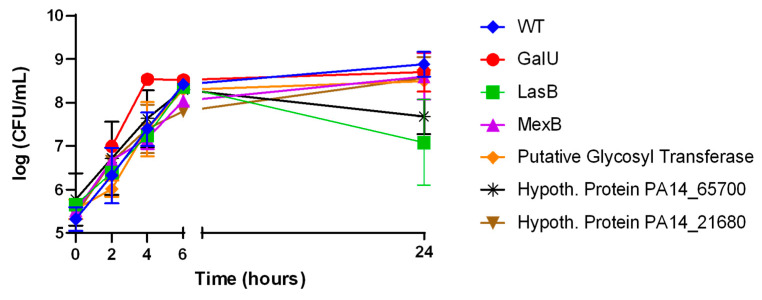
Growth curve of PA14 wildtype and phage-resistant mutants. Exponentially growing cultures were inoculated around a 5 × 10^5^ colony forming unit in 1mL of LB media. CFU were enumerated over time. The experiment was carried out in independent biological triplicate. Mean and standard deviation are presented here.

We carried out phenotypic characterization of each mutant in comparison to the PA14 wildtype with the purpose of determining the impact of transposon-mediated gene interruption on bacterial physiology. Growth curve analysis revealed normal exponential growth patterns (Figure 2), suggesting that the transposon insertion did not carry any fitness cost in actively replicating mutants. However, mutants carrying insertions in genes coding for a conserved hypothetical protein PA14_65700 and LasB elastase showed decreased viability in the stationary phase of growth, with over one log reduction in CFU for the LasB mutant (Figure 2).

**Figure 3:**
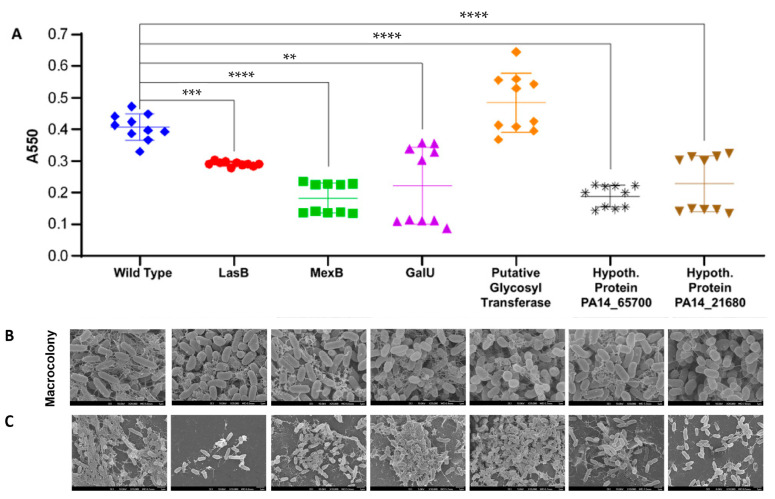
Biofilm formation in PA14 wildtype and phage-resistant mutants. (**A**) Quantitative analysis of static biofilm formation via crystal violet staining. Biofilm formation of *P. aeruginosa* wildtype and resistant mutants was quantified using crystal violet staining and spectrophotometry. Statistical analysis: Mann–Whitney test, *p* values < 0.01 (**), <0.001 (***) and <0.0001 (****). (**B**) Scanning electron microscopy (SEM) imaging of macrocolonies and (**C**) stationary phase cultures. Stationary phase culture or macrocolonies of *P. aeruginosa* wildtype and resistant mutants were either transferred or grown directly on nitrocellulose membranes placed on LB agar prior to being processed for SEM imaging.

*P. aeruginosa* has the ability to form biofilms under various conditions, which confers it with protection against antibiotics and, to a certain extent, shields the bacteria from phages. We quantified the formation of biofilm in all PA14 mutants and wildtypes using crystal violet staining on 24-h static biofilms. Interestingly, all mutants formed less biofilm except for the putative glycosyl transferase mutant (Figure 3A). These results were supported by scanning electron microscopy (SEM) imaging of macrocolonies and stationary phase cultures of the PA14 wildtype and mutants (Figure 3B,C). SEM images revealed a reduced propensity of mutants to form biofilms-like structures with a decrease in extracellular polymeric substances (Figure 3B,C).

Phage-resistant mutants of *P. aeruginosa* were previously shown to lose antibiotic resistance [27]. Specifically, *oprM* deletions mutants were shown to regain sensitivity to penicillin. OprM corresponds to the outer membrane protein part of the MexAB–OprM complex. Its loss abolishes the efflux pump’s ability to efflux drugs, restoring antibiotic sensitivity. We predicted that interruption of the inner membrane protein MexB would disrupt the assembly of the protein complex and lead to re-sensitization to penicillin antibiotics. To test our hypothesis, we quantified antibiotic susceptibility in all mutants and wildtypes. We determined a susceptibility profile to 15 common antibiotics across four mechanistically different classes using the Vitek 2 system (Table 1). As we predicted, the MexB mutant regained sensitivity to penicillin, including ampicillin and combinations of ampicillin/sulfabactam, piperacillin/tazobactam, as well as the antifolate combination of trimethoprim/sulfamethoxazole. The susceptibility to ampicillin alone was only classified as intermediate, probably due to beta-lactamases encoded by PA14. Unexpectedly, the GalU mutant displayed an intermediate level of resistance to ceftazidime compared to PA14 wildtype but remained sensitive to all the other cephalosporins.

Taken together, these results show that phage resistance was associated with phenotypic trades-off. While all mutants grew similarly to the wildtype during the exponential phase of growth, two of them (with an insertion in the genes coding for LasB and a hypothetical protein) showed decreased viability during the stationary phase. All mutants but one (putative glycosyl transferase mutant) formed less biofilms and extracellular polymeric substances. Additionally, and as predicted, the *mexB* mutant regained sensitivity to penicillin and antifolates.

### 2.3. Reduced Adsorption Is a Common Broad Resistance Mechanism to Phage Infection

**Figure 4:**
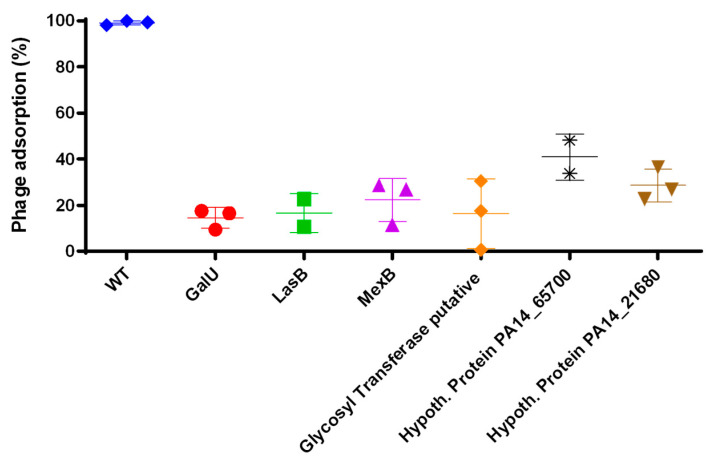
Quantification of phage adsorption on PA14 wildtype and resistant mutants. PA wildtype (WT) and all mutants were incubated with phages at MOI 0.01 for 5 min. Phages were recovered and enumerated. Adsorption was determined as the percentage of phage inoculum (see Section 4). The mean and standard deviation of three independent biological replicates is shown here.

We aimed to determine the underlying mechanism by which gene disruption led to phage-resistance in the mutants we identified. Deletion of the *galU* gene was previously shown to confer resistance to phage infection [28] via reduced adsorption of phages on the mutant membrane. *galU* mutants were devoid of O-antigen and synthesized a defective LPS core, which is required for phage adsorption [24,30]. A phage’s ability to infect bacteria depends on its ability to bind to their receptor, typically embedded in the bacterial membrane. Moreover, membrane alteration and reduced binding of phages on bacterial membrane receptors constitute the most common resistance mechanisms to infection [11]. Considering that the genes we identified here were involved in membrane synthesis or composition, we hypothesized that the membrane of the corresponding transposon mutants was altered. We hypothesized that phage adsorption was reduced on mutants with membrane alteration. To test our hypothesis, we carried out an adsorption assay in which we quantified phage attachment on the bacterial membrane in the PA14 wildtype and mutants (Figure 4). As expected, phage adsorption ability dropped below 15% on the *galU* mutant, consistent with what was reported in *P. aeruginosa* PA1, while nearing 100% on the PA14 wildtype. As we hypothesized, adsorption decreased below 50% on all mutants, dropping to 16% in *LasB* and the putative glycosyl transferase mutants, and 22% in the *mexB* mutant. These results point toward an altered membrane composition in all mutants preventing phage attachment and subsequent infection.

Having established that phage adsorption was significantly reduced in all mutants, we asked if resistance extended to other phages. We tested the susceptibility of the mutants to infection by nine different phages isolated from sewage targeting the *P. aeruginosa* PA14 wildtype. We carried out a lytic plaque assay and showed that all nine phages lysed the PA14 wildtype (Supplementary Figure S1) but did not form lytic plaque on any of the six mutants. These results suggest that the transposon-mutants are broadly resistant to different phages. It also suggests that these phages utilize similar membrane receptors.

## 3. Discussion

*P. aeruginosa* ranks among the critically important pathogens associated with antibiotic-resistant hospital-acquired infection worldwide. Therapeutic options to treat *P. aeruginosa* infections are increasingly limited. Phage therapy has shown promising results in the treatment of antibiotic-resistant *P. aeruginosa* infections. *P. aeruginosa*, like all bacteria, can develop resistance to phages. The development of PT requires a clear understanding of these resistance mechanisms. Transposon libraries offer a convenient tool to interrogate the entire genome of an organism in the search for genes involved in the mechanisms of phage infection and resistance. Here, we report the phenotypic screening of the complete *P. aeruginosa* PA14NR transposon mutant library for genes involved in resistance to phage infection. We identified six mutants that did not support the formation of lytic plaques and did not show a decrease in CFU over time when exposed to phages. The genetic analysis of these mutants revealed the interruption of the six following genes: *galU*, *mexB*, *LasB*, a putative glycosyl transferase (PA14_66110), and two conserved hypothetical proteins (PA14_21680 and PA14_65700). Insertion in lasB and PA14_21680 led to reduced viability during the stationary phase of growth, while other mutants were unaffected. All mutants showed a reduced ability to form biofilm except for the putative glycosyl transferase mutant (PA14_66110). Electron microscopy analysis further revealed that these mutants produced less extracellular polymeric substance. Phage adsorption was significantly reduced in all mutants, suggesting that reducing the ability of phages to attach to their host is a common mechanism of resistance to phage infection in *P. aeruginosa*. It also suggests that the genes identified somehow play a role in either membrane synthesis or phage binding to membrane receptors. Finally, all mutants showed broad resistance to different natural phages.

One of the most common ways bacteria have evolved to resist phages relies on the modification of membrane receptors that phages utilize to attach and invade their host. Many *P. aeruginosa* phages recognize LPS or type IV pili as receptors [25]. Modifications to these conserved elements confer phage resistance, albeit to the detriment of virulence. Several studies have reported phage-resistant *P. aeruginosa* LPS or pili mutants with attenuated virulence [25,26,27]. For example, chromosomal deletion of a loci containing *galU* was previously reported in the *P. aeruginosa* PA1 strain selected for phage resistance. GalU converts glucose-1-phospate into UDP-D-Glucose, a predominant sugar in the LPS outer core. Consistent with our findings, PAO1 *galU* deletion mutants lacked O-antigen and synthesized a deficient LPS, leading to reduced phage adsorption [18,22,24]. Glycosyltransferases are another important group of enzymes which add monosaccharides onto sugars, proteins, and lipids backbones, and are involved in LPS biosynthesis [28]. A transposon insertion in the gene PA_5001 of the *P. aeruginosa* PA1 strain, which codes for a putative glycosyl/glucosyltransferase, showed resistance to phage infection [22]. The putative glycosyl transferase we identified, encoded by PA14_66110, is only a distant orthologue of PA_5001, with only 5% identity and 17% similarity at the amino acid level, but shows a similar phenotype.

Another membrane protein complex and multidrug efflux transporter, MexAB–OprM, was shown to serve as a receptor of phage OMKO1 in the *P. aeruginosa* PAO1 strain. Deletion of the gene encoding the outer membrane protein, OprM, conferred resistance to infection by phage OMKO1. Accordingly, our results suggest that the MexB protein, which anchors the complex in the inner membrane, is also required for phage infection in PA14. In absence of MexB, the anchoring of the protein complex in the inner membrane is likely compromised. The mexB gene is located upstream of the OprM gene in an operon, and the expression of OprM could be affected in the mexB mutant. Deletion of OprM was also shown to restore sensitivity to antibiotics [21]. We observed a similar trade-off in the MexB mutant, which regained sensitivity to penicillin and antifolate antibiotics. Surprisingly, we did not identify OprM nor the mexA mutants in the course of our screening. This could be due to a growth defect associated with the interruption of these genes, which could have led to these mutants not passing our screening threshold of growth in the presence or absence of phages.

Interestingly, our results show that interruption of the gene coding for the elastase B enzyme (LasB, PA14_16250) also conferred resistance to phage infection. LasB has never been associated with phage-resistance. LasB is a secreted metalloprotease and virulence factor of the *P. aeruginosa* PA14 strain [23,29]. It has been shown to play an important role in the pathogenicity of *P. aeruginosa* during host infection and is involved in biofilm formation [30]. Expectedly, the LasB mutant showed impaired biofilm formation. The expression of the *lasB* gene is regulated by the quorum sensing (QS) transcription systems las and rhl [31]. The MexAB–OprM complex has been implicated in the secretion of the quorum-sensing molecule homoserine lactone, which in turn modulated the expression of LasB. The major role of proteases in *P. aeruginosa* virulence in various infection models is thought to involve tissue penetration (Twining et al., 1993; Tang et al., 1996) [32,33]. *P. aeruginosa* proteases include LasB, LasA, and alkaline protease AprA. We tested the effect of *lasB* interruption on elastolytic activity, that is, the ability to degrade elastin, and observed only a marginal reduction (Supplementary Figure S2). This could be explained by the fact that *P. aeruginosa* elastolytic activity is the sum of the activity of LasA, LasB, and AprA, which cannot be distinguished in the Elastin Congo red assay. LasB is expressed as precursor P that is exported to the periplasm and processed into PI followed by PII plus a propeptide component, P20 [34,35,36]. P20 binds noncovalently to PII and inhibits its degradative activity. P20-PII is secreted and P20 degraded, releasing an active LasB. A *lasB* deletion mutant was made in PAO1 and shown to exhibit significantly decreased bacterial attachment and extracellular matrix linkage in biofilm, associated with decreased biosynthesis of rhamnolipids [30]. It is tempting to speculate that inactivation of LasB leads to modification of LPS composition, which in turn impairs phage adsorption, thereby conferring resistance. However, the dominant-negative effect we observed and the precise mechanism by which it confers resistance to phage infection remains unclear and requires further studies. Moreover, we could not conclusively attribute the phage-resistance phenotype to LasB as complementation with the native LasB gene failed to restore the sensitive phenotype. We also identified two mutants with transposon insertion in genes PA14_21680 and PA14_65700, coding for two hypothetical proteins with no functionally annotated homologs nor previous association with phage resistance. Finally, 19 out of 71 of the mutants found in our intermediate hits carry insertion in genes coding for membrane components or involved in membrane composition. These results further reinforce the importance of the membrane as a target-space in phage–bacteria interactions.

In summary, the newly identified genes galU, mexB, LasB, and others appear to contribute to phage resistance through various mechanisms involving membrane composition and biofilm formation. GalU is essential for LPS biosynthesis, and its disruption leads to deficient LPS, reducing phage adsorption. MexB, part of the MexAB–OprM efflux pump, likely influences phage susceptibility by affecting membrane receptor availability and antibiotic resistance, as phage OMKO1 utilizes this complex for entry. LasB, a metalloprotease involved in biofilm formation and virulence, might alter LPS composition and reduce phage binding when inactivated. The putative glycosyl transferase (PA14_66110) and hypothetical proteins (PA14_21680 and PA14_65700) may play roles in membrane synthesis or modification, further limiting phage attachment. Understanding these genes’ contributions to phage resistance can guide the development of phage therapy strategies, potentially through the design of phage cocktails that target multiple pathways, or the combination of phage therapy with antibiotics to counteract resistance mechanisms.

Areas of future research include elucidating how a mexB mutant affects the expression and functionality of the MexAB–OprM complex, as this could reveal critical insights into membrane transporter assembly and function, phage resistance mechanisms, and antibiotic susceptibility. Additionally, we could employ high-density transposon libraries or other advanced methods like TraDIS [31] (Transposon Directed Insertion-site Sequencing) to help identify additional genes involved in resistance to phage infection, providing a broader understanding of the genetic basis of resistance. Furthermore, exploring the intermediate resistant population—mutants that did not prevent lytic plaque formation but exhibited clear resistance—could uncover novel resistance pathways and potentially identify secondary factors or compensatory mechanisms that contribute to phage resistance.

Our results show that transposon mutant libraries and forward genetic screens of dominant-negative effects constitute an efficient method to identify genes involved in phage–host interactions and resistance. A broader and deeper understanding of the mechanisms essential to phage infections and resistance mechanisms developed by bacteria are prerequisites to designing efficacious phage cocktails and preventing the emergence of resistance.

## 4. Materials and Methods

Bacterial strains, phage isolates, and culture conditions. The ordered, nonredundant library of *P. aeruginosa* PA14 transposon insertion mutants library was obtained from https://pa14.mgh.harvard.edu/cgi-bin/pa14/home.cgi. *P. aeruginosa* PA14 wildtype and mutants were all grown in Lysogeny Broth (LB) medium. Mutants were grown and stored in LB broth supplemented with gentamicin at 15 µg/mL in 96-well plates (Greiner Bio-One, Singapore, Singapore). The phage vB_Pae_SG_Moreira_PyoP2 was isolated from the commercial Pyo Cocktail (Eliava Institute, Tbilisi, Georgia), while phages vB_Pae_SG_WM_Sew_P3, vB_Pae_SG_WM_Sew_P14, vB_Pae_SG_WM_Sew_P15, vB_Pae_SG_WM_SEW_P16, vB_Pae_SG_WM_Sew_P23, vB_Pae_SG_WM_Sew_P27, Sew P5, and Sew P17 were isolated from Singapore. The complete phage genome sequences were deposited into GenBank with the following accession numbers: PP716131, vB_Pae_SG_Moreira_PyoP2; PP740684, vB_Pae_SG_WM_Sew_P3; PP740685, vB_Pae_SG_WM_Sew_P14; PP740686, vB_Pae_SG_WM_Sew_P15; PP740687, vB_Pae_SG_WM_SEW_P16; PP740688, vB_Pae_SG_WM_Sew_P23; PP740689, vB_Pae_SG_WM_Sew_P27.

Phage DNA extraction, genome sequencing, and annotation. Phage DNA was extracted following the method used in [34]. Extracellular genetic material was removed from the phage enrichment by nuclease digestion (10 µg/mL DNase I, 10 µg/mL RNase A, 5 mM MgCl_2_) at 37 °C for 1 h. Phage virions were precipitated using polyethylene glycol (10% *w*/*v* PEG8000, 1 M NaCl) at 4 °C overnight. The virions were collected by centrifuging at 13,000× *g* for 15 min and resuspending the pellet with 1 mL SM buffer (0.1 M NaCl, 8 mM MgSO_4_, 50 mM Tris-HCl, 0.01% gelatin, pH 7.5). Phage virions were lysed with EDTA, SDS, and Proteinase K (20 mM EDTA, 0.5% SDS, 50 µg/mL Proteinase K). An equal volume of 25:24:1 phenol–chloroform–isoamyl alcohol was added to the sample, and the mixture was centrifuged at 10,000× *g* for 3 min. The upper phase, containing gDNA, was collected and transferred to a fresh tube containing 1 volume of isopropanol and 0.1 volume of 3M sodium acetate (pH 5.2), and incubated at room temperature for 30 min. Precipitated phage gDNA was collected by centrifuging at 10,000× *g* for 10 min. The DNA pellet was washed with 70% ethanol and air-dried. The gDNA was resuspended in 50 µL nuclease-free water. The DNA samples were cleaned up using the Zymo Genomic DNA Clean & Concentrator™, following the manufacturer’s instructions. The gDNA was eluted in 30 µL of elution buffer. Sequencing was performed on the Illumina HiSeq X platform using the TruSeq Nano DNA Library preparation kit. The reads obtained were trimmed using Trimmomatic [35] to remove adapters, contaminations, or low-quality sequences. Contigs were assembled using Shovill [36]. Assembled sequences were annotated using Pharokka [37], and phage taxonomy was determined using PhaGCN [38] and PhageAI [39].

PA14NR library phenotypic screen. The PA14 nonredundant transposon library is a comprehensive collection of mutant strains of *Pseudomonas aeruginosa* PA14, each with a single gene disrupted by a transposon insertion. This library is designed to cover the majority of nonessential genes in the PA14 genome, each gene being disrupted only once. The complete library of 5459 transposon insertion was screened in stepwise manner for mutants resistant to phage infection. Briefly, 96-well plate cultures of mutants were freshly prepared by inoculating frozen glycerol stocks from library master plates into freshly prepared 96-well plates containing 200 µL of LB using a sterilized 96-pin replicator. The replicator was sterilized in-between each inoculation by immersion in 99% ethanol followed by flaming. The mutant plates were incubated at 37 °C overnight. The next day, each plate was sub-cultured in duplicate by inoculating 2 µL of overnight culture into 96-well plates freshly filled with 100 µL of LB. One control plate was left untreated and received an additional 100 µL of LB per well, while one plate was inoculated with phages at a final concentration of 10^8^ PFU/ML in 100 µL of LB. Both control and phage-infected plates were incubated at 37 °C for 4 h. Each plate was then resuspended manually using a multichannel pipette, and OD600 was recorded using a BioTek Synergy 4 plate reader (Singapore, Singapore). Hits were defined as phage-treated mutants exhibiting OD600 ≥ 70% of nontreated corresponding control. Growth-deficient mutants (OD600 ≤ 0.07) were eliminated. The primary screen was conducted in a single biological replicate, and hits were validated in a secondary screen carried out in two independent biological replicates using the same criteria.

Lytic plaque assay. Lytic plaque formation was determined on soft agar overlays. Briefly, 24-well LB agar plates were inoculated with an overnight culture of each mutant, resuspended in 300 µL of soft agar. Upon solidification, 2 ×0^5^ PFU of phages in 2 µL were spotted at the center of the soft agar layer and air-dried. Plates were incubated at 37 °C for 24 h, and pictures were taken. The assay was carried out twice in biological replicates.

Phage kill-kinetic. Overnight cultures grown from a single colony were sub-cultured and grown to log-phase (OD_600_ = 0.6) at 37 °C. A total of 10^7^ CFU/mL were inoculated in 1 mL LB and treated with phages at a final concentration of 10^8^ PFU/mL (MOI = 10) and incubated at 37 °C on an orbital shaker at 220 rpm. CFU were enumerated by serial dilutions on LB agar plates at regular time intervals. The experiment was carried out in two independent biological replicates.

Phage adsorption assay. Exponentially growing cultures were centrifuged at 13,000× *g* for 1 min and cell pellets washed with LB medium and resuspended to a final concentration of 10^8^ cfu/mL. Phages were then added at a final concentration of 10^6^ PFU/mL. The mixture was incubated at 37 °C for 5 min. Samples were then filtered with a 0.22 µm filter, and supernatants were collected for phage titer determination by enumeration of PFU via plaque assay. Adsorption ability (%) is represented as [(Titer inoculated -Titer in supernatant/Titer inoculated)] × 100%.

Macrocolony assays. Macrocolonies were obtained by inoculating single colonies from each mutant in LB broth with 50 µg/mL Gentamycin at 37 °C, 200 rpm culture overnight (~16 h). Overnight culture was sub-cultured to log-phase. Nitrocellulose (NC) membrane was UV-sterilized for one hour on each side then placed on LB agar. A 5 µL quantity of log-phase cultures were spotted onto sterile NC membrane on LB agar and incubated at 37 °C overnight until the formation of a macrocolony.

Biofilm formation assay. Biofilm formation in 48 h on polystyrene 96-well plates was quantified using crystal violet staining and spectrophotometry, as previously described [40]. Biofilm formation of stationary phase cultures was prepared as follow for imaging purposes: nitrocellulose membranes were incubated together with the different mutants in LB broth (starting inoculum ≈ 105 CFU/mL) for 15 h in 48-well plates. After which, LB broth was displaced with ice-cold 2.5% glutaraldehyde to the wells, and membranes were fixed overnight at 4 °C.

Scanning Electron Microscopy. Phosphate buffer solution (1×PBS) was used to wash the fixed membranes; buffer was changed twice. Washed membranes were kept in PBS at 4 °C. A total of 1% osmium tetroxide was used to post-fix the membranes, and ascending concentrations of ethanol were used to dehydrate the samples. The membranes were then critical-point-dried, stuck on carbon stubs, and coated with 25 nm of gold. A Field Emission Electron Microscope (EM) at the National University of Singapore was employed to view the samples.

Genetic complementation. The wildtype versions of each gene identified in the mutants were cloned into the broad-host range pBBR1MCS-2 overexpression vector [41], except for the oprM complementation, where we used pBR1MCS-3. pBR1MCS-2 and pBR1MCS-3 carry a kanamycin-resistant cassette and a tetracycline-resistant cassette, respectively. The plasmids were introduced in their corresponding mutant following the protocol of [42]. Transformant were selected on kanamycin-containing LB agar plates. Single colonies were picked and culture was expanded for further characterization of the complemented mutants.

Elastase activity assay. Elastolytic activity was determined by the elastin–Congo Red assay [43]. The assay reflects the elastolytic enzymatic activity in a cell-free supernatant capable of digesting elastin from the elastin–Congo Red complex, allowing the Congo Red dye to be released from the complex, producing color that can be measured at an absorbance of 495 nm (A495).

Antibiotic susceptibility testing. The antimicrobial susceptibility profiles of both mutants and wildtype PA14 were determined using the automated Vitek^®^ 2 Compact system. Commercially available AST-GN79 cards (BioMérieux, Singapore, Singapore) were used according to the manufacturer’s recommendations to determine MIC and the susceptibility profile against a panel of antibiotics.

## Figures and Tables

**Table 1:** Antibiotic susceptibility profile of the PA14 wildtype and resistant mutants. S = Susceptible; I = Intermediate; R = Resistant based on MIC; nd = not determined. All mutants showed high levels of resistance to gentamycin when compared to the wildtype. This is due to the gentamicin resistance cassette integrated alongside the transposon in the genome of all mutants in the PA14NR library.

	WT	GalU	LasB	MexB	Putative Glycosyl Transferase	Hypoth. Protein PA14_65700	Hypoth. Protein PA14_21680
Ampicillin	R	R	R	I	R	R	R
Ampicillin/Sulbactam	R	R	R	S	R	R	R
Piperacillin/Tazobactam	S	nd	nd	S	S	S	S
Trimethoprim/Sulfamethoxazole	R	R	S	S	R	R	R
Cefazolin	R	R	R	R	R	R	R
Cefoxitin	R	R	R	R	R	R	R
Ceftazidime	S	I	S	S	S	S	S
Ceftriaxone	R	R	R	I	R	R	R
Cefepime	S	S	S	S	S	S	S
Meropenem	S	S	S	S	S	S	S
Amikacin	S	S	S	S	S	S	S
Gentamicin	S	R	R	R	R	R	R
Tobramycin	S	S	S	S	S	S	S
Ciprofloxacin	S	S	S	S	S	S	S
Nitrofurantoin	R	R	R	R	R	R	R

## Data Availability

Data is contained within the article and Supplementary Materials.

## Supplementary Materials

The following supporting information can be downloaded at: https://www.mdpi.com/article/10.3390/ijms25137009/s1.
