# Supplementary material for: Screening of the PA14NR Transposon Mutant Library Identifies Genes Involved in Resistance to Bacteriophage Infection in Pseudomomas aeruginosa
Source: Int J Mol Sci. 2024 Jun 26;25(13):7009. doi: 10.3390/ijms25137009 (PMC11241198; doi:10.3390/ijms25137009)
Supplement: Supplementary file 1 [file ijms-25-07009-s001.zip › ijms-3025157-supplementary.pdf]

Supplementary material

Mutant ID	Gene Locus	Gene Name	Gene Description	Bacteriophage susceptibility phenotype in mutants	Bacteriophage susceptibility phenotype in revertants
52640	PA14_38350	galU	UTP-glucose-1-phosphate uridylyltransferase (GalU)	Resistant	Sensitive
31938	PA14_16250	lasB	Elastase (LasB)	Resistant	Resistant
6501	PA14_05540	mexB	RND multidrug efflux transporter (MexB)	Resistant	Sensitive
56681	PA14_66110	-	Putative glycosyl transferase	Resistant	Sensitive
28071	PA14_21680	-	Conserved hypothetical protein	Resistant	Sensitive
31922	PA14_65700	-	Conserved hypothetical protein	Resistant	Sensitive

Supplementary Table S1. Susceptibility and Resistance phenotype of transposons mutants and revertant. The wildtype version of each gene identified in the mutants were cloned into the broad-host range pBBR1MCS-2 overexpression vector carrying a Kanamycin resistant gene. The plasmids were introduced in their corresponding mutant following and transformants were selected on kanamycin containing LB-agar plates. Bacteriophage susceptibility was determined using lytic plaque assay.

Gene Locus	Gene Description	Gene name	Cellular component	Biological process
PA14_27850	putative GTP cyclohydrolase	queF	cytoplasm	oxidoreductase activity
PA14_18550	alginate biosynthesis protein Alg44	alg44	membrane	biofilm formation
PA14_56830	Insulin-cleaving metalloproteinase outer membrane	icmP	membrane	na
PA14_42640	translocation protein in type III secretion	pscT	membrane	protein targeting
PA14_58810	conserved hypothetical protein	-	membrane	ion transport
PA14_37640	conserved hypothetical protein	-	membrane	electron transfer
PA14_49810	hypothetical protein	-	membrane	na
PA14_31310	hypothetical protein	-	membrane	na
PA14_43400	potassium-transporting ATPase, A chain	-	membrane	potassium transmembrane transporter
PA14_01380	putative cytochrome c oxidase assembly factor	-	membrane	transferase activity
PA14_41630	putative cytoplasmic membrane-associated protein	-	membrane	na
PA14_15080	putative iron-regulated membrane protein	-	membrane	na
PA14_48340	putative membrane protein	-	membrane	transmembrane transport
PA14_00550	putative membrane protein	-	membrane	na
PA14_27270	putative permease	-	membrane	na
PA14_64890	putative permease of ABC branched chain amino acid transporter	-	membrane	transmembrane transport
PA14_69060	putative permease of ABC transporter	-	membrane	transmembrane transport
PA14_37730	TonB dependent receptor	-	membrane	transmembrane transport
PA14_07580	glycerol-3-phosphate acyltransferase PlsY	-	membrane	phospholipid metabolism
PA14_39000	conserved hypothetical protein	-	membrane	porin activity
PA14_23070	glucose-6-phosphate 1-dehydrogenase	zwf	na	carbon metabolism
PA14_61400	malate:quinone oxidoreductase	mqo	na	carbon metabolism
PA14_08350	anthranilate phosphoribosyltransferase	trpD	na	biosynthetic process
PA14_41560	assimilatory nitrate reductase	nasA	na	oxidoreductase activity
PA14_25970	cobalamin biosynthetic protein CobN	cobN	na	biosynthetic process
PA14_24910	molybdenum cofactor biosynthesis protein A2	moeA2	na	biosynthetic process
PA14_41170	NADH-dependent enoyl-ACP reductase	fabI	na	Fatty acid and lipid metabolism
PA14_36000	propionate catabolism operon regulator	prpR	na	transcription regulation
PA14_65480	thiosulfate sulfurtransferase	rhdA	na	sulfurtransferase activity
PA14_08560	tyrosyl-tRNA synthetase 2	tyrZ	na	tRNA aminoacylation
PA14_12530	conserved hypothetical protein	-	na	na
PA14_60520	conserved hypothetical protein	-	na	na
PA14_54270	conserved hypothetical protein	-	na	na
PA14_38010	conserved hypothetical protein	-	na	na
PA14_56870	conserved hypothetical protein	-	na	protein binding

Gene Locus	Gene Description	Gene name	Cellular component	Biological process
PA14_16210	conserved hypothetical protein	-	na	na
PA14_53260	hypothetical protein	-	na	na
PA14_03110	hypothetical protein	-	na	na
PA14_13360	hypothetical protein	-	na	na
PA14_18050	hypothetical protein	-	na	na
PA14_51160	hypothetical protein	-	na	na
PA14_31450	hypothetical protein	-	na	na
PA14_52080	hypothetical protein	-	na	na
PA14_12680	possible short-chain dehydrogenase	-	na	Fatty acid and lipid metabolism
PA14_53700	probable ring-cleaving dioxygenase	-	na	na
PA14_52190	probable RNA methyltransferase	-	na	RNA methyltransferase
PA14_30760	putative acetyltransferase	-	na	acetyltransferase activity
PA14_09630	putative acyl-CoA dehydrogenase	-	na	helicase activity
PA14_12630	putative ATP-dependent helicase	-	na	helicase activity
PA14_56530	putative beta-lactamase	-	na	beta-lactamase activity
PA14_70600	putative DNA-binding protein HU family	-	na	DNA binding
PA14_42870	putative enzyme	-	na	na
PA14_35670	putative glycosyl hydrolase	-	na	hydrolase activity
PA14_36420	putative histidine kinase	-	na	signal transduction
PA14_10240	putative hydrolase	-	na	carbon metabolism
PA14_56200	putative lipoprotein	-	na	protein binding
PA14_24880	putative lipoprotein	-	na	protein binding
PA14_72250	putative metalloprotease	-	na	metal ion binding
PA14_15820	putative N-acetylglucosamine-6-phosphate deacetylase	-	na	hydrolase activity
PA14_54920	putative non-ribosomal peptide synthetase	-	na	catalytic activity
PA14_04140	putative oxidoreductase, FAD-binding	-	na	catalytic activity
PA14_22060	putative patatin family protein	-	na	lipid metabolism
PA14_08300	putative phage-related protein, tail component	-	na	protein binding
PA14_48160	putative sensor/response regulator hybrid	-	na	signal transduction
PA14_26330	transcriptional regulator, AraC family	-	na	transcription regualtion
PA14_01980	transcriptional regulator, LysR family	-	na	transcription regualtion
PA14_17440	tRNA pseudouridine synthase D	truD	na	RNA modification
PA14_30430	thiosulfate sulfurtransferase	-	na	small molecule transport
PA14_32060	transcriptional regulator XylS	xylS	na	transcription regualtion
PA14_20190	copper ABC transporter, periplasmic copper-binding protein	nosD	periplasm	transport
PA14_16000	50S ribosomal protein L19	rplS	ribosome	translation

Supplementary Table S2. Intermediate hits. The genetic background information of 71 intermediately resistant mutants was retrieved from PA14NR database. Cellular component and Biological process information was retrieved from gene ontology, functional and pathway analysis on the Pseudomonas genome DB (https://pseudomonas.com/). The 6 mutants that displayed totally resistant phenotype in our secondary screen were removed from that table.

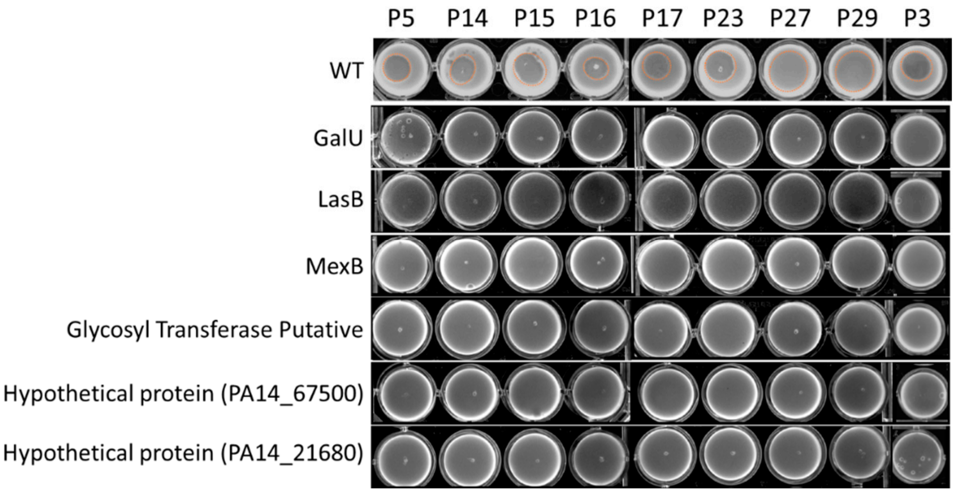

Supplementary Figure S1. Cross-resistance to bacteriophage infection in transposon-mutants.

All mutants were subjected to Lytic plaque formation assay using 9 different natural phages isolated from Singapore sewages against wildtype PA14 strain of *P. aeruginosa*. None of the mutants supported plaque formation, indicating cross-resistance to all the phages.

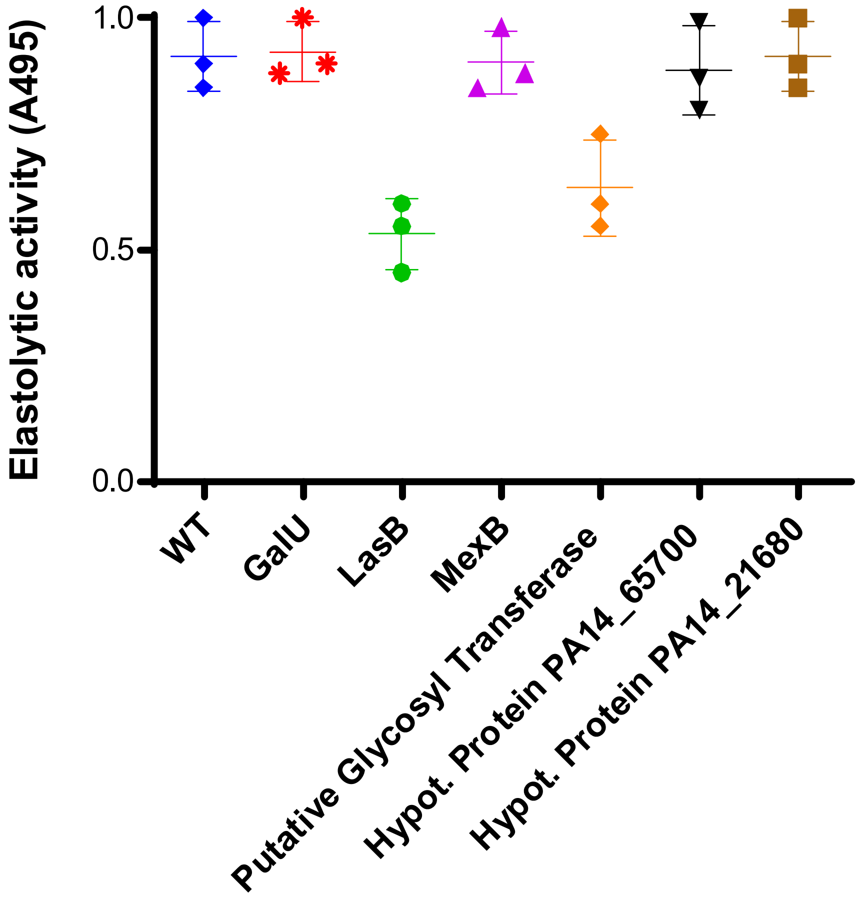

Supplementary Figure S2. Elastolysin Activity in *P. aeruginosa* WT and mutants. Elastolytic activity of all mutants was determined by elastin-Congo Red assay. The assay reflects the elastolytic enzymatic activity in cell-free supernatant capable of digesting elastin from the elastin-Congo Red complex, allowing the Congo Red dye to be released from the complex producing color that can be measured at absorbance of 495 nm (A495).
